# A systematic review of the effectiveness of self-management interventions in people with multiple sclerosis at improving depression, anxiety and quality of life

**DOI:** 10.1371/journal.pone.0185931

**Published:** 2017-10-11

**Authors:** Tara Kidd, Nicola Carey, Freda Mold, Sue Westwood, Maria Miklaucich, Emmanouela Konstantara, Annette Sterr, Debbie Cooke

**Affiliations:** 1 School of Health Sciences, Faculty of Health and Medical Sciences, University of Surrey, Guildford, Surrey, United Kingdom; 2 School of Psychology, University of Surrey, Guildford, United Kingdom; Columbia University Medical Center, UNITED STATES

## Abstract

**Background:**

Self-management interventions have become increasingly popular in the management of long-term health conditions; however, little is known about their impact on psychological well-being in people with Multiple Sclerosis (MS).

**Purpose:**

To examine the effectiveness of self-management interventions on improving depression, anxiety and health related quality of life in people with MS.

**Method:**

A structured literature search was conducted for the years 2000 to 2016. The review process followed the PRISMA guidelines, and is registered with PROSPERO (no. CRD42016033925).

**Results:**

The review identified 10 RCT trials that fulfilled selection criteria and quality appraisal. Self-management interventions improved health-related quality of life in 6 out of 7 studies, with some evidence of improvement in depression and anxiety symptoms.

**Conclusion:**

Although the results are promising more robust evaluation is required in order to determine the effectiveness of self-management interventions on depression, anxiety and quality of life in people with MS. Evaluation of the data was impeded by a number of methodological issues including incomplete content and delivery information for the intervention and the exclusion of participants representing the disease spectrum. Recommendations are made for service development and research quality improvement.

## Introduction

Multiple Sclerosis (MS) is a chronic, degenerative, autoimmune disease of the central nervous system that affects approximately 2 million people globally [1]. It is characterised by an early onset of disease, diagnosed in young adults typically between 20–40 years, and is associated with a relatively normal length of life expectancy [2]. Symptoms including sensory and motor loss, fatigue, pain and depression [3, 4], are often unpredictable in frequency, severity, and trajectory [5]. Moreover, untreated or poorly managed symptoms can lead to severe and potentially life threatening complications [6]. Consequently, people with MS face a multitude of physical, mental and emotional challenges on a daily basis [2, 7].

Self-management is a potential approach that may mitigate the symptoms associated with MS. Self-management interventions (SMI) are a relatively new phenomenon in the health research field but are increasingly seen as key to effective management of long-term conditions [8]. Self-management can be defined as: *‘the individual’s ability to manage the symptoms*, *treatment*, *physical and psychosocial consequences and lifestyle changes inherent in living with a chronic condition’* [9]. There is now substantial evidence of health benefits following self-management interventions in long term conditions such as diabetes, arthritis, and heart disease [10]. It is recognised that self-management may be relevant for people with MS, and there is some evidence of its success in promoting skills for managing specific clinical outcomes such as fatigue or medication adherence [11–13]. Self-management interventions also offer an opportunity to address skills for promoting psychological well-being [10]. However, relatively little is known about their effect on anxiety and depression in people with MS. This is important given that people with MS tend to experience higher levels of anxiety and depression compared to the general population [14, 15].

Patten et al., [16] for example reported the 12 month prevalence of depression for people with MS was 25.7% compared to 8.9% in the general population. It has been estimated that the lifetime prevalence of anxiety is 37% and depression is as high as 50% in people with MS [17, 18]. It is likely these figures are understated for depression, as health care professionals often attribute depressive symptoms to the disease [19]. Depression symptoms have wide ranging implications for the health and well-being of people with MS, including increased fatigue, pain, decreased adherence to medication, immune functioning, exacerbation of the disease and reduced quality of life (QOL) [20, 21].

People with MS not only have significantly poorer QOL than the general population, but also in comparison to those diagnosed with other long-term illnesses such as epilepsy, diabetes, rheumatoid arthritis and irritable bowel disease [22]. Importantly, studies have increasingly demonstrated that depression symptoms independently predict MS specific health-related QOL (MS-QOL) [23] and general health-related QOL (HRQOL) [24], over and above clinical markers such as neurological disability, or levels of fatigue. Self-management interventions that address depression and anxiety symptoms may therefore result in improved health outcomes and HRQOL.

Currently, there is little robust evidence to support the effectiveness of SMI on reducing depression and anxiety symptoms and improving HRQOL. One review conducted by Rae-Grant et al., [7] examined self-management interventions in neurological disorders, including MS, Parkinson’s disease, and migraine. Inclusion criteria for self-management interventions included self-managed exercise programs, motivational interviewing and goal setting, group and/or individual self-management sessions, internet-based self-management strategies, telephone prompting strategies, lay-led self-management, and self-managed wellness programs. Additionally, the small number of studies (n = 9), the heterogeneity of study design (e.g. randomised control trials (RCT), pre and post, qualitative) and outcomes measured (e.g. self-efficacy, pain, perceived control, QOL) makes it difficult to compare the efficacy of the different treatment approaches. The authors concluded that self-management interventions significantly improved QOL outcomes in people with MS and showed promise in the treatment of long-term neurological conditions.

More recently, Kuspinar and colleagues [25] conducted a meta-analysis examining the combined effects of different psychological interventions on HRQOL among people with MS. They reported a small but statistically significant cumulative effect size (0.24) across self-management interventions designed to improve HRQOL. Again, the studies included were very varied in their focus, used different interventions and incorporated different aims. Consequently, the studies within each of these categories may not have been similar enough to examine their combined effect in the meta-analysis, and may partly account for the small effect size reported.

The objective of this review is to build on existing work by focusing on randomised control trials (RCT) of self-management interventions aimed at improving depression, anxiety and/or quality of life in people with MS exclusively, in order to allow for greater ease of comparison across studies. We describe the active components of the interventions to try and identify what works well, for whom and under what conditions. Our review question is: Are self-management interventions effective at improving depression, anxiety symptoms and quality of life in people with MS?

## Method

A systematic review of the literature was conducted with evidence sourced from 2000 to 2016. The review process followed the PRISMA guidelines [26], and is registered with PROSPERO (no. CRD42016033925).

### Search strategy

**Fig 1:**
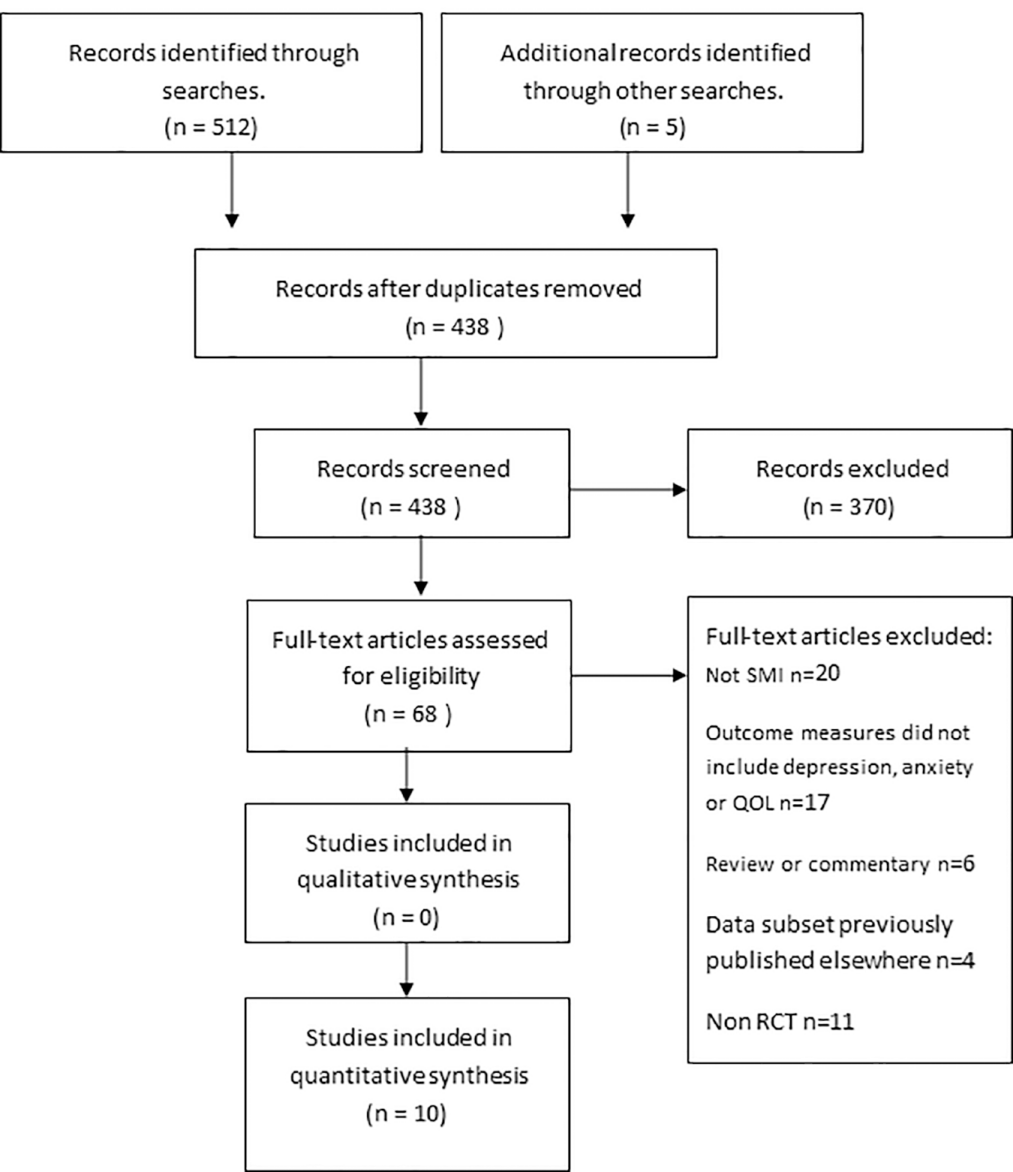
PRISMA flow diagram of the search process.

Targeted searches of specialist databases were conducted using the following index/MeSH (Medical Subject Heading) and strings of keyword terms, (MS) plus (intervention, self -management, self-care, self-monitor, self-help) plus (depression, anxiety, or QOL). Databases included the Cochrane Database of Systematic Reviews, MEDLINE, EMBASE, CINAHL and PsycINFO (see table in S1 Table: Example search string using Medline). Search results were exported into EndNote X7 software (Thomas Corporation) and duplicates removed before titles and abstracts were screened in relation to the inclusion/exclusion criteria. Citations were screened by one reviewer (DC) and were checked independently by the two other reviewers (TK & EK). All three reviewers confirmed the eligibility of the identified studies. Any disagreements about possible inclusion were resolved by a group discussion. The search process is shown in Fig 1.

### Eligibility criteria

Results of searches were checked against pre-defined inclusion criteria:

1. Randomised Control Trials (RCTs) that reported quantitative outcome data on one or more well-being measures of depression, anxiety or QOL following a self-management intervention in people with MS.
2. Interventions had to contain self-management components aimed at improving the well-being of participants. These included learning and practising particular skills (behavioural, cognitive) to improve and maintain well-being.
3. RCT’s focusing on adult participants only (aged ≥18 years) with a MS diagnosis of any type (e.g. primary progressive, secondary progressive, relapsing-remitting, progressive relapsing).
4. Interventions where adults with MS were a comparison group, among other patient groups, were also included.
5. Studies published in a peer reviewed publication and available in English.

Table 1 provides an overview on the SMI components of the studies included in this review. We focused on RCTs as this type of study design generally supports greater validity and causal inference [27].

**Table 1:** Descriptive information for each study conducted in the systematic review.

First author (year)	Sample size	Age Mean±SD	EDSS Mean ±SD	Intervention	Duration and Frequency	Follow up	Control	SMI	Primary outcome of study	Well-being outcome measure(s) (Effect size)	Summary of findings
Barlow et al., **[28]**	78 (I) 64 (WLC)	48.2 ±10.1 50.7 ±11.7	Not present	Chronic disease self-management course	Weekly 2hr sessions x 6.	4 month 12 month	WLC	B SE PS GD R ST	Self-Efficacy	HADS Depression (ES 0.25) Anxiety (ES 0.14)	Treatment group had improved SM self-efficacy compared to control. NSD reported for anxiety or depression.
Bombardier et al., **[29]**	70 (I) 60 (WLC)	47.5 (41–54) 45.0 (40.5–52.0)	Not present	Motivational interview and telephone counselling	1 motivational interview (60-90mins) x 5 telephone counselling sessions (30 minutes).	PI	WLC	MI B	Health promotion	SF-36 MCS (ES x)	Treatment group had significant improvements in health promotion behaviours and MCS QOL compared to controls (p<0.05).
Ehde et al., **[30]**	75 (I) 88 (C)	51.0±10.1 53.2±10.0	≤4 (I) 25.3% (C) 26.1% 4.5–6.5 (I) 61.3% (C) 62.5% ≥7 (I) 13.3% (C) 11.4%	Remote delivery self-management course vs. education program.	8 x telephone sessions (45–60 minutes).	PI 6 month 12 month	Telephone delivered education program group.	C B	Fatigue Pain Depression	PHQ-9 Depression (ES -0.14) SF-8 MCS (ES 0.03) PCS (ES 0.01)	Both groups had ≥50% symptom reduction in 1 or more primary outcomes. NSD between SM and education groups on depression or QOL outcomes. However, only the SM group had significant improvement in PCS PI and at 6 months.
Ennis et al., **[31]**	32 (I) 30 (C)	45±9 46±8	91% (0–6) 97% (0–6)	Health promotion education program	8x sessions (3 hours).	PI	WLC	SE PS GE	Health promotion behaviours Self-efficacy	SF-36 Physical (ES -0.21*) Social (ES-0.39) Role physical (ES -0.76) Role emotional (ES -0.11) Mental health (ES -0.82***) Fatigue (ES -0.26) Pain (ES -0.19) General health (ES -0.53**)	Treatment group had significant improvements in health promotion behaviours, self-efficacy, and physical function, mental and general health QOL compared to WLC (p<0.05).
Finlayson et al., **[32]**	89 (I) 92 (WLC)	56.0±9.0 (Pooled sample)	Not presented	Remote delivery fatigue management program.	6x group sessions (70 mins)	PI 6 weeks 3 month 6 month	WLC	PS ST DM SS	Fatigue	SF-36 (ES x)	Intervention group had significant improvement in fatigue and role physical QOL following the intervention compared to control group (p<0.05). Significant improvements found in 6 out of 8 QOL subscales for pooled data (p<0.05).).
Graziano et al., **[33]**	41 (I) 41 (C)	42.3 ±8.5 38.3 ±10.1	All participant 1–5.5	Cognitive behavioural group intervention	4 x sessions (2 hours).	PI 6 month	Usual care	C B R	QOL Self-Efficacy Depression	MSQOL-54 (ES -0.40*) CES-D (ES 0.29)	Intervention group had significant improvements in QOL (p<0.05) and self-efficacy in comparison to the control group at 6 months. NSD for depression outcomes.
O’Hara, et al., **[34]**	73 (I) 96 (C)	52.5±11.2 50.4±10.4	Not presented	Self-management program	2x sessions (2 hours).	6 month	No SMI control	B GD	Mobility HR-QOL	SF36 Mental health (ES -0.23*) Pain (ES -0.12) Physical role (ES 0.16) Physical function (ES -0.07) Role emotional (ES 0.02) Social function (ES -0.13) Vitality (ES -0.27*) General health (ES -0.13)	Treatment group had significantly better mental health and vitality QOL than control group at 6 months (p<0.05).
Khan et al., **[35]**	49(I) 52(C) 24 (IP I) 25 (OP I)	49.5 (8.64) 51.1 (9.64)	0–3 14.3% (I) 23.1% (C) 3.5–6.0 55.1% (I) 61.5% (C) 6.5+ 30.6% (I) 15.4% (C)	Individualised MD rehabilitation program.	5 day inpatient rehabilitation program. OR 2 to 3x outpatient sessions for 6 weeks (30 mins).	12 month	WLC	E R ST	Functional independence	GHQ-28 Anxiety (ES 0.01) Depression (ES -0.05).	Treatment group improved in functional independence measures but NSD for anxiety and depression relative to control group.
Miller et al., **[36]**	83 (I) 84 (C)	48.1 (9.7) 48.1 (9.1)	Not presented	Remote delivery self-management program	12 month access to enhanced messaging service.	PI	Usual care	ST	Sickness impact profile Self-efficacy	EURO-QOL (ES <0.01)	No differences were reported between the enhanced group and the regular treatment group.
Moss-Morris et al.,**[37]**	23 (I) 17 (C)	40.14±17.76 41.81±11.81	Not presented	Remote delivery self-management program	8-10x online sessions (25–50 mins), plus 3 x telephone support sessions (30–60 mins).	PI	Usual care	C B	Fatigue	HADS Anxiety (ES 0.87***) Depression (ES 2.14***)	Treatment group had significant reductions in fatigue, depression and anxiety (p<0.05).

KEY PI Post Intervention, SM Self-management, WLC waiting list control, NSD no significant difference, HADS Hospital Anxiety and Depression Scale, QALY’s Quality of adjusted life years, PHQ-9 Patient Health Questionnaire, GHQ-28 General Health Questionnaire, SF-8/36 Short Form Health Survey, MCS Mental Composite Score, PCS Physical Composite Score. SMI components (adapted from Steed, Cooke and Newman, [47]) C Cognitive, ST Skills training, B behavioural, PS problem solving, GE general education, GD general discussion, R relaxation, E exercise, DM decision making, SS social support, MI motivational interview.

Classification of effect size *small, **medium ***large, where p<0.05, x impossible to calculate effect size based on reported results.

To examine the effectiveness of SMIs at improving well-being, as broadly as possible, no restrictions were placed on MS disease severity, type of MS, duration of disease, presence of comorbid conditions, adult age, gender, ethnicity or type of control group used. Studies were excluded if participants were under 18 years or were studies based on purely educational interventions. Studies that used subsets of data published in full elsewhere were not included, thus to prevent any duplication of data.

Searches across all database and additional searches yielded n = 517 results. After applying the inclusion/ exclusion criteria n = 68 remained. Full text articles were retrieved and on closer inspection did not fulfil the review eligibility. A final total of 10 articles were eligible and included in the analysis (see Fig 1).

### Data extraction

Data extraction was conducted by one researcher using a pre-designed data extraction form (DEF) reflecting the core study areas, together with data on the methods and results necessary to support critical appraisal. The DEF included the basic outline of the evidence under study such as aims, primary/secondary outcomes, sample, intervention content, length of follow-up, analysis methods, results, intervention effectiveness and study limitations. Reference lists of all primary studies, qualitative studies and review articles on the topic were searched for additional references. Data extracted from each study were entered into a summary table to enable comparison of study and participant characteristics, and results (Table 1). We chose to examine the outcomes taken from the final follow up for several reasons. Firstly, there was no obvious comparable time point across studies due to heterogeneity. Second, previous research suggests the effects of behaviour change may require longer duration to pass before psychological benefits are likely experienced [38].

### Measure of effect size

Hedges adjusted g calculation [39] was used to examine the effect of each self-management intervention on depression, anxiety or QOL outcomes. This is obtained by taking the difference in the mean change score in the outcome (pre- and post-intervention) between an intervention and a control group and then dividing by the initial pooled standard deviation (SD). Cohen’s criteria was used to interpret the size of the effect, where small is 0.2, medium is 0.5 and large is 0.8. An effect was statistically significant if p≤0.05.

### Strength of evidence assessment of studies

Each study was analysed for bias using the Cochrane Risk of Bias Tool [40]. The risk of bias tool assesses seven domains which are sequence generation, allocation concealment, blinding of participants and personnel, blinding of outcomes assessed, treatment of incomplete data, selective outcome reporting and other risks of bias. The risk of bias in each subcategory was classified as high, low or unclear. The assessment of bias was conducted independently by two authors (TK & DC) and decisions were compared and discussed to achieve consensus (Table 2).

**Table 2:** Risk of bias.

	Random sequence generation	Allocation concealment	Blinding of participants and personnel	Blinding of outcome assessment	Incomplete outcome data addressed	Selective outcome reporting	Other bias	Decision
**Barlow et al., [28]**	Low	Low	High	High	Low	Low	Low	Low risk
**Bombardier et al., [29]**	Low	Low	High	High	Low	Low	High	Moderate risk
**Ehde et al.,[30]**	Low	Low	Low	Low	Low	High	High	Low risk
**Ennis et al.,[31]**	Low	Unsure	High	High	Low	Low	Low	Low to moderate risk
**Finlayson et al., [32]**	Low	Low	Low	High	Low	Unclear	High	Low to moderate risk
**Graziano et al., [33]**	Low	Unsure	Low	High	Low	Low	High	Low to moderate risk
**Khan et al., [35]**	Low	Unsure	High	High	Low	Low	Low	Low to moderate risk
**Miller et al., [36]**	Low	Low	High	High	High	Low	Low	Moderate risk
**Moss-Morris et al., [37]**	Low	Low	High	Unsure	Low	Low	High	Low to moderate risk
**O’Hara et al., [34]**	Low	Low	High	High	High	Low	Low	Moderate risk

## Results

### Study characteristics

Ten RCTs were included in the review. Of these studies, 5 evaluated the impact of the intervention on depression [28, 30, 33, 35, 37] 3 on anxiety [28, 35, 37] and 7 on QOL [29–34, 36] as an outcome. Psychological variables were predominantly assessed as a secondary outcome of the study (n = 7) [28, 29, 31, 32, 35–37]. Sample sizes varied from 40 to 181. The total and mean numbers of participants were 1,286 and 128.6. The range of participants’ ages from the studies was 25–81 years, with the majority having a mean age in the 40’s or 50’s. Approximately 70% of each sample population was female. Anti-depressant and anxiety medication use was not reported in any study. Nine out of ten studies reported a physician diagnosis of MS, but only four studies included Expanded Disability Status Scale (EDSS) scores [30, 31, 33, 35]. The majority of participants had ambulatory function without aid for at least 100m distance. Time since diagnosis was on average 10 years. Studies were relatively heterogeneous with respect to the components applied to the SMI. Behavioural components, e.g. goal setting, were the most common (n = 6) [28–30, 33, 34, 37]; whereas only 3 studies incorporated a CBT element into the intervention [30, 33, 37]. Studies were conducted in an outpatient context (n = 3) [31, 33, 35]; a local community setting (n = 2) [28, 34]; or at home (n = 5) [29, 30, 32, 36, 37]. Half of the studies were delivered in a group setting (n = 5) [28, 31–34] and the remaining half on an individual basis [29, 30, 35–37]. Five studies delivered their self-management intervention remotely either online (n = 2) [36, 37], or telephone (n = 3) [29, 30, 32]. Follow up ranged from 2 weeks to 12 months post intervention.

### Anxiety

Three studies examined the impact of a SMI on anxiety outcomes. Anxiety was measured using the Hospital Anxiety and Depression Scale (HADS) [41] (n = 2) [28, 37], and the General Health Questionnaire (GHQ-28) [42] (n = 1) [35]. Of these, 2 used waiting list control groups as a comparison [28, 35]. One study reported an improvement relative to a non-treatment comparison group [37]. Significant reductions in anxiety symptoms were found 2 weeks post intervention for this 8 week, interactive, online CBT based SMI (Effect Size = 0.87 p<0.05).This was also the only study to have people with MS who reported clinically significant anxiety symptoms at baseline (mean HADS score = 8.26, SD 4.31). The two remaining studies utilised different SMI approaches, the first was a generic chronic disease SMI delivered over 6 weekly group sessions, comprising problem solving, general discussion, and education [28]. The second was an individualised, goal-orientated rehabilitation targeted intervention that focused primarily on physical aspects of rehabilitation and utilised goal setting self-management components [35]. Neither study reported a significant difference in anxiety symptoms relative to a waiting list control group at 12 month follow-up.

### Depression

Five studies considered the impact of SMI on depressive symptoms. Of these only 2 used a waiting list control group as a comparison [28, 35], the remaining studies compared the intervention to usual care [33, 37] or an education intervention [30]. The tools used to measure depression were varied: HADS [41] (n = 2) [28, 37]; Center for Epidemiological Studies Depression Scale (CES-D) [43] (n = 1) [33], GHQ-28 [42] (n = 1) [35]; and the Patient Health Questionnaire (PHQ-9) [44] (n = 1) [30]. Three studies reported improvements in depression scores over time in the intervention groups [30, 33, 37], but only one of these reported significant improvement related to a control group [37] at 2 weeks post intervention (Effect Size = 2.14 p<0.05). It is notable that it is this study that also achieved significant improvement in anxiety symptoms relative to its control group (Effect Size = 0.87; see section above). A significant improvement in depression symptoms were also reported by another study delivering the intervention by telephone, with longer follow up of 6 and 12 months; however, the intervention group did not perform significantly better than the education treatment comparison group [30]. Finally, a trend toward reduction in depression symptoms relative to a usual care control group was reported 6 months post intervention for a group-based SMI (p = 0.051) [33]. A commonality between these studies is that they all utilised CBT principles in developing the intervention. Studies reporting non-significant results were group-based, goal directed, non-MS specific [28, 33, 35], or participants did not report depressed symptoms at baseline [28, 35].

### Quality of life

The impact of SMI on QOL in people with MS was assessed in 7 studies [29–34, 36]. Out of these 7, 6 studies reported significant improvement in QOL over time relative to a control group. SMIs that incorporated CBT and/or behavioural components, such as goal setting, demonstrated the most improvement in QOL outcomes. Four studies were delivered remotely (online n = 1, phone n = 3), of these 3 reported significant improvements in HR-QOL [29, 30, 32]. Improvements in QOL were reported immediately post intervention and up to 12 months later. Overall, effect sizes were small across studies for QOL outcomes ranging from to 0.23–0.82, (all p<0.05).

The most widely used tool was the short form health survey (SF-36) [45], a generic HR-QOL outcome measure, comprising 8 subscales (physical functioning, role physical, role emotional, mental health, vitality, bodily pain, general health perception and social functioning), that are combined to form 2 composite scores indicating overall physical and mental HR-QOL. Two studies examined physical and mental composite scores [29, 30] and 3 studies examined individual sub-scales [31, 32, 34].

Significant improvement in mental composite QOL items was the most common outcome [29, 31, 34], and the physical composite QOL items to a lesser degree [31, 32], all p<0.05, (see Table 1). Only one study examined MS-specific QOL, and despite not achieving a significant reduction in depression symptoms, this SMI was effective in improving QOL over time [33]. Several restrictions were placed on participant entry in four of the studies reporting positive findings that were related to disability severity, pain, and fatigue. This meant that only people with MS who had mild to moderate symptoms were able to participate [29, 30, 32, 33].

## Discussion

To our knowledge this is the first review that has specifically focused on examining the effectiveness of SMIs at improving symptoms of depression, anxiety and quality of life in people with MS. This review highlights the paucity of high quality controlled trials of SMIs (n = 10) designed to improve psychological well-being in the MS population and identifies a significant gap in the research literature. While results initially appear encouraging and are in line with existing work [7, 25], there is insufficient evidence to determine the exact extent to which SMIs led to improvements in anxiety, depression and HRQOL in people with MS.

Delineating which SMI components worked best was impeded by lack of detailed information describing the intervention and its constituent components and interactions [46, 47]. Overall, SMIs that incorporated aspects of CBT and behavioural components such as goal setting were most commonly associated with improvements in psychological well-being. This was especially true for improvements in anxiety and depression symptoms [33, 37]. There is a reasonable body of evidence demonstrating the efficacy of CBT in treating depression and anxiety symptoms in people with MS [48, 49]. Moreover, CBT and behavioural components appeared successful in improving QOL outcomes [48] which also seemed to the case in this current review. However, it is impossible to determine the direction of effect based on the small number of studies that included depression and/or anxiety and QOL in this review. Reducing symptoms of anxiety and depression may improve QOL directly or indirectly supporting the need for underpinning theoretical frameworks when designing interventions.

We purposefully reported on the final follow-up assessment following SMI, as there is some evidence that psychological benefits may not always present immediately following an intervention [38]. Furthermore, identifying SMIs that deliver longer lasting effects offer the greatest benefit to people with MS and will be more appealing to service providers considering implementation [50]. We encountered multiple assessment time points across studies, from immediately post intervention up to 12 months after. Overall results were positive with evidence that improvements in QOL were maintained over time, which is consistent with the wider literature [51]. The longer lasting effects on reduction in depression and anxiety symptoms following an SMI are less conclusive, as the only study reporting improvement relative to a control group was directly following intervention completion [37]. Although beyond the scope of this review, studies examining SMI should consider the impact of timing on follow-up assessment, as different outcomes may be associated with different time frames which could have practice implications [50, 52].

Narrow inclusion criteria also meant that results could not be generalised to a wider MS population. Several studies recruited only those individuals who were functioning at a moderate to high level, with little physical disability, and who did not report severe depression or anxiety symptoms [29, 30, 32, 33, 37]. Correspondingly, studies have shown that people with MS who are less physically restricted are much less likely to experience psychological distress [53, 54]. It follows that if individuals are functioning at an optimal level prior to the intervention there is likely to be a ceiling effect; thereby making it very difficult to demonstrate any positive effects of the intervention as there is little room for improvement. This may explain the small magnitude of effect reported for improvements in QOL, and contribute to the non-significant findings reported for depression and anxiety in the current review. Future studies should widen selection criteria to prevent reporting bias towards younger people at the early stages of the disease trajectory.

A promising finding was that four out of five SMIs reported improvements in either depression, anxiety or QOL outcomes following remote delivery of treatment, three of which were compared to a control group. Increasingly technology is being developed around patient-centred treatment that improves health and well-being across a wide array of medical and long-term conditions [55, 56]. Developing technology to deliver SMIs remotely could offer easier access for people who may be otherwise prohibited from attending treatment [57]. People with MS with greater disability who experience higher levels of depression and social isolation may benefit from remote delivery methods, as research suggests they are less likely to participate in face to face therapy or group programs [14, 58]. Increased knowledge of patient-related facilitators and barriers of success of SMIs can inform the development of tailored interventions based on individual patient profiles and preferences, including face to face, remote delivery, or blending these [59].

The role of caregivers also needs to be considered in future SMI development as they are often instrumental to successful symptom management in people with MS [60]. Caregiver involvement was not considered in any of the studies reviewed. There is mounting evidence of psychological distress in the carers of people with MS, but there has been little development of services or provisions to ease caregiver burden in this group [61]. Future work should focus on developing an intervention to improve psychological well-being in both people with MS and carers.

In addition to the methodological strengths and weaknesses of the studies reviewed we must consider the limitations of this review. Despite applying rigorous criteria heterogeneity was still evident in terms of design, delivery, and outcome. This was illustrated by the complexity of interventions and the variations between studies on SM intervention components. Relatedly, multiple self-report questionnaire measures of outcome were included in this review that differed in their sensitivity to measure change [54], and further impeded cross-study comparison. Finally, a descriptive approach was utilised for data analysis rather than meta-analysis. Meta-analysis was considered inappropriate for this review due to the heterogeneity in the design, measures and outcomes reported.

## Conclusion

The small number of RCT SMIs available to include in this review reveals a significant gap in the research literature. Further RCT studies with larger more inclusive samples are needed to allow sub-group analysis to determine what SMI components work best for people with different types of MS, as well as exploring the optimal method of delivery. Furthermore an important progression in the SMI field would be to develop agreed guidelines for researchers and clinicians on best practice in designing and reporting studies in this area (e.g. [62]). This could include agreeing on a core set of outcome measures to be used in quantitative studies; recommendations for the type of information reported e.g. disease type/severity in study results; inclusion of participants representing the disease spectrum; recommendations for reporting the content and delivery of an intervention, its component parts, and follow-up period.

## Supporting information

S1 Table Example search string using Medline. (PDF)

## Abbreviations

MS: multiple sclerosis
HRQOL: health-related quality of life
NICE: the national institute for health and care excellence
IAPT: improving access to psychological therapies
PRISMA: preferred reporting items of systematic reviews
RCT: randomised control trial
CBT: cognitive behavioural therapy
SMI: self-management intervention
DEF: data extraction form
WLCG: waiting list control group
HADS: hospital anxiety and depression scale
CES-D: center for epidemiological studies depression scale
GHQ-28: general health questionnaire
PHQ-9: patient health questionnaire
SF-36: short form health survey
ES: effect size
UK: United Kingdom
