# Supplementary material for: A systematic review of the effectiveness of self-management interventions in people with multiple sclerosis at improving depression, anxiety and quality of life
Source: PLoS One. 2017 Oct 11;12(10):e0185931. doi: 10.1371/journal.pone.0185931 (PMC5636105; doi:10.1371/journal.pone.0185931)
Supplement: S1 Table — (PDF) [file pone.0185931.s001.pdf]

Supporting information

S1 Table. Example search string using Medline

Platform EBSCO Database Medline Limits 2000-2016; Human.

S1	self*care.ab.ti
S2	self*manag*.ab.ti
S3	self*monitor*.ab.ti
S4	self*help.ab.ti
S5	OR/S1-S4
S6	(MH "Multiple Sclerosis+")
S7	(MH "Myelitis")
S8	(MH "Myelitis, Transverse")
S9	(MH "Demyelinating Autoimmune Diseases, CNS")
S10	(MH "Multiple Sclerosis, Chronic Progressive")
S11	(MH "Multiple Sclerosis, Relapsing-Remitting")
S12	multiple N6 Sclerosis ti.ab
S13	OR/S6-S12
S14	S5 and S13

Key
Ti– title word
Ab – abstract word
MH – Main index/ MeSH term
